# Community experiences with police and implications for public health: A focus group study

**DOI:** 10.1371/journal.pgph.0003123

**Published:** 2024-06-11

**Authors:** Moutasem A. Zakkar, Se Lim Jang, Fariba Kolahdooz, Sarah Deck, Christina Gillies, Adrian Wagg, Sangita Sharma

**Affiliations:** 1 Department of Medicine, Indigenous and Global Health Research Group, Faculty of Medicine & Dentistry, College of Health Sciences, University of Alberta, Edmonton, Alberta, Canada; 2 Provincial Population and Public Health, Alberta Health Services, Edmonton, Alberta, Canada; 3 Department of Medicine, Division of Geriatric Medicine, Faculty of Medicine & Dentistry, College of Health Sciences, University of Alberta, Edmonton, Alberta, Canada; University of Colorado Denver - Anschutz Medical Campus: University of Colorado - Anschutz Medical Campus, UNITED STATES

## Abstract

Interactions with the police can impact an individual’s short and long-term physical, mental, and social wellbeing, as well as levels of violence and unrest within a community. As such, this study aimed to explore experiences with the police among individuals experiencing socioeconomic disadvantages in Edmonton, Canada. For this qualitative study, participants (n = 39) were recruited from an emergency shelter for women, a drop-in community organization supporting individuals experiencing socioeconomic disadvantages, and a centre providing settlement support for newcomers to Canada. During the recruitment process, the research information sheet, including the scope and goals of the study, was presented, and participants who had any experience with the police were recruited. Each participant joined one of seven focus groups, during which experiences with the police were discussed; data from the focus groups were analyzed utilizing thematic analysis. Factors that contributed to satisfactory experiences with the police included the police demonstrating responsiveness and compassion, as well as an individual’s understanding of police work. Factors that contributed to unsatisfactory experiences included the experiences of discrimination, stigmatization, and disrespect during interactions with the police. Participants suggested that community-police relationships could be improved by police being less judgemental and suspicious in their approach, undergoing regular training in sociocultural sensitivity, and being more open in their communication and community outreach. Overall, adopting a less discriminatory and more empathetic approach within a police force is essential for creating and maintaining a positive community-police relationship. By considering the socioeconomic context of people’s behaviours and actions, police can better support the health and wellbeing of individuals and communities.

## Introduction

Policing refers to actions carried out by the police with the intention of maintaining law, order, and conformity to societal norms [1–3]. Attitudes and perspectives held by a community and its police toward policing, including the characteristics and outcomes of interaction with police, are referred to as the community-police relationship [4, 5]. In 2019, one in three Canadians reported having an interaction with the police [6]. The number of encounters increased substantially for people experiencing socioeconomic disadvantages characterized by poverty, low income, and limited opportunity for social mobility [7], including people experiencing housing instability, people living with mental health conditions, Indigenous community members, and visible minorities [6, 8, 9].

Several proxy indicators have been used to represent the community-police relationship from the perspective of community members, including whether people believe that the police are a legitimate authority [10], as well as people’s trust in [11], satisfaction with [12], respect for [13], and willingness to cooperate with the police [11]. Procedural justice theory asserts that the perceived fairness of policing encounters affects a person’s satisfaction with and appreciation of the police [14–16]. Elements of a police encounter necessary for procedural justice include police neutrality, police treating individuals with dignity and enabling expression of differing perspectives, and police prioritizing the wellbeing of individuals and society [11, 14]. Other factors affecting people’s satisfaction and relationship with the police include police performance in terms of crime prevention and keeping community safety [14, 17], police compassion as expressed by being supportive, caring, and friendly with people [18], and police responsiveness, which represents the police’s ability to prioritize and address people’s needs and concerns [19].

Each year, 20% of Canadians experience mental health conditions [20], and more than 235,000 Canadians experience homelessness [21, 22]. People experiencing poverty, social inequity, food insecurity, material deprivation, and low social capital are at a higher risk for mental health conditions [23–25]. Individuals experiencing mental health conditions might have higher rates of police encounters compared to people not experiencing these conditions [26], and if mental health conditions are combined with homelessness, the odds of police encounters become even higher [27]. In 2012, 20% of Canadians who interacted with the police had mental health conditions and were four times more likely to be arrested [28]. From 2000 to 2017, 42% of the cases in which people were killed during police encounters involved people with mental health conditions [29, 30].

Policing itself is a social determinant of health, albeit infrequently recognized and less understood [31–33]. Unsatisfactory encounters with the police (e.g., forceful, disrespectful, or discriminatory) can have detrimental effects on a person’s short and long-term physical, mental, and social wellbeing, behaviour, and mortality, as well as levels of community violence [34–36], and can result in individual and inter-generational trauma and community unrest [34–36]. Discriminatory policing against racial and ethnic minorities may also be associated with depression, anxiety, gastrointestinal symptoms, headaches, and binge drinking [34, 37]. More generally, discrimination may increase stress, leading to worsened cardiovascular diseases, obesity, mental health conditions, and weakened immune systems [38].

The impact of policing on health depends on policing practices within communities and moderating factors across all levels (i.e., individual, community, and systemic), including ethnic background, gender, poverty, housing instability, and mental health [36]. A growing body of research has started to explore the impact of policing on health through factors such as satisfaction with the police and experiences of discrimination [39–41]. Many police departments worldwide, including Edmonton Police Service (EPS), have also begun exploring ways to improve community-police relationships with individuals experiencing socioeconomic disadvantages and visible minorities [11, 42].

This study was part of the Caring and Responding in Edmonton (CARE) research project [43] based in Edmonton, Alberta, Canada. The project explored healthcare access and utilization and patient experience of people experiencing socioeconomic disadvantages to identify opportunities for improvement in the healthcare system. Part of this project focused on experience with the police of socio-economically disadvantaged people in recognition that this experience is a determinant of health.

## Methods

### Setting, design and data collection

This study employed a qualitative descriptive design [44, 45]. This methodology seeks to identify explicit themes in the qualitative data with minimum interpretation of the data by the researchers [45]. The methodology is often used to explore a phenomenon before employing more complex qualitative methodologies such as phenomenology or grounded theory [44].

Purposive sampling was used to recruit participants from an emergency shelter for women, a drop-in community organization providing shelter, support and mental health counselling for people experiencing homelessness, and a centre providing settlement support and mental health counselling for newcomers to Canada. The organizations were in different geographic locations in Edmonton. The majority of the clients of the organizations are people experiencing socioeconomic disadvantages. The selection of the organizations was made based on the project objectives and in consultation with a community advisory board (CAB) comprised of community, grassroots, governmental, cultural, and religious organizations in Edmonton, and it also included organizations providing wraparound services for newcomers and low-income families.

Project posters were placed in these organizations to raise awareness of the study and included the project contact information for people interested in participation. The research team presented the project to the organizations’ staff, explained its goals and research focus, and engaged with the organizations’ clients in rapport-building activities such as lunches, playing games, and crafting. The research team then presented the project, its expected outcomes and benefits, and data collection methods to organizations’ clients. Nearly 80% of the 50 participants who were approached and invited by the organizations’ staff agreed to participate. Reasons for not participating included low interest and limited availability.

During the recruitment process, the research information sheet, including the scope and goals of the study, was presented, and only people who had experience with the police were recruited. Following the disclosure of the study’s objectives and methods, participants provided written informed consent. The consent form also provided information on voluntary participation and withdrawal at any time during the study. Participants who agreed to participate were put in groups of five to six people and invited to one of the respective meetings. For participants’ convenience, participants in any group were clients of the same organization. After the meetings, participants received $15 gift cards to grocery stores or coffee shops, and there were no other benefits.

Focus groups led by two research team members (FK, SJ) were used to collect data. Participants (n = 39) joined one of seven focus groups, with an average of five participants per group and two groups per organization. The groups lasted for an average of 60 minutes. All participants attended the full respective meetings.

The meetings took place on the participating organizations’ premises. Before starting the discussion, the team members provided background information about the research group, and its previous work and research agenda and explained the project goals. One team member moderated the discussion while the other took notes. A set of open-ended questions were carefully designed to help the moderators start the discussions (Table 1). These questions were reviewed by the CAB. Participants were asked to describe experiences with the EPS. The moderator also encouraged participants to express participants’ perspectives on improving policing experience. Social workers working at the participating organizations were available to provide counselling to participants to relieve any stress that might be associated with the shared experiences.

**Table 1:** Discussion questions.

Questions
What positive interactions have you had with the Edmonton Police Service?
What negative interactions have you had with the Edmonton Police Service?
What do you think can be done to improve the situation when interacting with the police?

The number of focus groups was determined by carefully reviewing the collected data and researcher notes after each discussion until data saturation was reached [46, 47]. With participants’ permission, the discussions were audio-recorded, transcribed verbatim, and de-identified to protect confidentiality. Participants were asked to respect the confidentiality of information discussed in the group and were informed that because multiple participants would be in the meeting, confidentiality could not be guaranteed. Demographic data were also collected using an electronic record form in REDCap (version 8.1.1), including age and gender, ethnic background, and the highest level of completed education.

To ensure research rigour, the Consolidated Criteria for Reporting Qualitative Research (COREQ) [48] were followed (S1 Table). Researchers also engaged in several reflective discussions to maintain high levels of objectivity and accuracy when presenting and interpreting participants’ views.

### Data analysis

**Fig 1:**
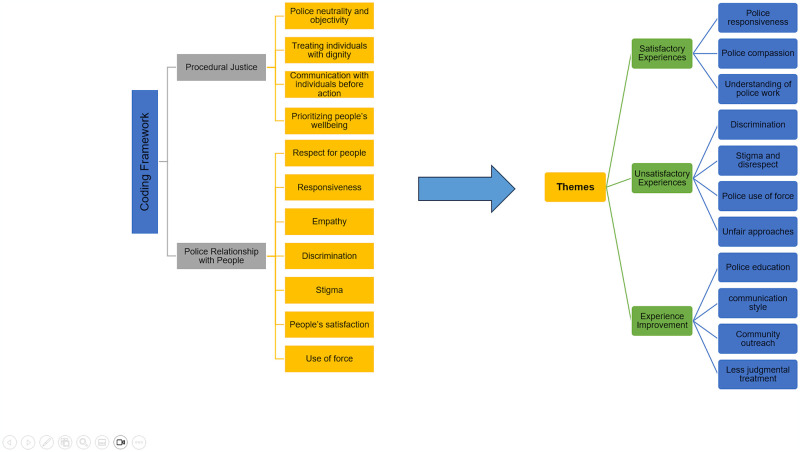
Community experiences with police and implications for public health: A focus group study.

This study used deductive thematic analysis. A coding framework (Fig 1) was created, *a priori*, based on procedural justice theory, incorporating other factors of police experiences identified through the literature review [11, 14, 17]. Guided by the coding framework, data were carefully read to determine prominent concepts, which were then used to identify and code the main themes, which were all part of the coding framework. The coding process was rigorous and iterative, including several rounds of reading, coding, and categorizing data. Two authors (MZ and SJ) conducted the coding process independently and resolved disagreements with the help of another author (FK). The accuracy of the codes was verified independently by the other authors. Qualitative data analysis was performed using NVivo Pro version 12 (QSR International Pty Ltd, 2018). Descriptive statistics of the demographic data were produced using SAS Software version 9.4 (SAS Institute Inc., 2013).

### Ethics

Ethical approval was obtained from the Research Ethics Board at the University of Alberta and the Northern Alberta Clinical Trials and Research Centre (PR00069624).

## Results

Nearly half of the participants (48.72%) were between 30–50 years of age, and 43.59% were ≥50 years of age; 61.54% were women, 46.15% were Indigenous; 20.51% were European, and 25.64% were Asian, African or Middle Eastern. One-third of the participants (33.33%) had a post-secondary degree or diploma, and one-third (35.90%) had less than or some high-school education. A quarter of the participants (28.21%) were residents of a women’s emergency shelter, and 46.15% were clients of an organization providing support for people experiencing socioeconomic disadvantages (Table 2).

**Table 2:** Characteristics of focus groups participants (n = 39).

Characteristics	n (%)
**Organization**	
Supporting people experiencing socioeconomic disadvantages	18 (46.15)
Supporting newcomers to Canada	10 (25.64)
Providing emergency shelter for women	11 (28.21)
**Age (years)**	
18–29	3 (7.69)
30–50	19 (48.72)
50 or more	17 (43.59)
**Gender**	
Male	15 (38.46)
Female	24 (61.54)
**Ethnicity** *	
Indigenous	18 (46.15)
African or Middle Eastern**	5 (12.82)
Asian	5 (12.82)
European	8 (20.51)
No response	3 (7.69)
**Education**	
Less than high school	7 (17.95)
Some high school	7 (17.95)
High school diploma	5 (12.82)
Some post-secondary	7 (17.95)
Post-secondary degree or diploma	13 (33.33)

* Participants could select more than one response

** These two levels were combined due to the low numbers of corresponding participants.

We identified three main themes to represent the participants’ experiences with the police: 1) perceived satisfactory experiences, 2) perceived unsatisfactory experiences, and 3) suggestions for improving experiences. These main themes were further divided into subthemes to represent more specific insights into policing, which are illustrated using direct quotations. Some of the quotations are presented in the main text, while others are listed in a table to avoid cluttering the main text.

### Theme 1: Perceived satisfactory experiences

Some participants expressed having satisfactory experiences with the police. Factors that contributed to these experiences were police responsiveness, police compassion, and people’s understanding of police work.

### Police responsiveness

This sub-theme denotes participants’ perception of the police’s readiness in responding to situations. One participant described how the police intervened during a violent encounter with an ex-partner:

“The police were there when I needed them. Someone called 911 when I was getting punched out by my ex, and if it wasn’t for that, he probably would have got away with killing me.”

(Participant 7)

Another participant expressed appreciation for how the police responded to a call reporting the participant’s attempted suicide (Table 3, #1), and another described how the police helped the participant get evidence of domestic violence for the court (Table 3, #2).

**Table 3:** Participants’ quotes.

**Theme 1: Perceived satisfactory experiences**		
**Subtheme**	**#**	***Participant quote***
Police responsiveness	1	*“They came after I attempted suicide when my son called 911*, *and I am really willing to thank Edmonton Police Service*. *All Canada*, *you know*, *cause*, *like everybody*, *they have a holiday*, *but Edmonton police don’t have a holiday*. *They are really nice*.*” (Participant 9)*
	2	*“I recently left a domestic violence situation*. *For my own safety*, *I actually had to get out of Edmonton*. *And when I was in [another city]*, *we filed an emergency protection order*, *and they [the police in the other city] wanted—because we’re going to court- they wanted to show a pattern of abuse*. *So*, *I had to get in touch with the Edmonton police force*, *and they were extremely helpful*.*” (Participant 12)*
Police compassion	3	*“I got pulled over*, *and I got a [driving under the influence of alcohol]*… *But he actually called another cop to come park my car instead of impounding it*. *Not only did he save me a lot of heartache and argument*, *but it was our only vehicle at the time*, *so my old man could still go to work*, *the kids*, *like we would have been so [improper word]*. *So*, *to have him dispatch another vehicle to come*, *and then they parked it*, *so we didn’t have to worry about time*, *you know*, *paying for the parking or anything so” (Participant 1)*
**Theme 2: Perceived unsatisfactory experiences**		
**Subtheme**	**#**	***Participant quote***
Use of force	4	*“-Participant 4*: *the only time I’ve ever got beat from a cop was*, *well*, *a few times*, *but I deserved it*. *I spit in the cop’s face*. *You end up getting beat for that*. *I deserved it*. *-Participant 2*: *But maybe it’s not fair if it’s an underage kid that’s intoxicated to get beat up by a cop*.*”*
	5	*“They pulled me out of the car*, *they say you can step out of the car*, *they pulled me behind the car and started stomping on my head*, *they knocked me out*. *Yeah*, *that’s the only time I got knocked out*. *They pepper-sprayed me*, *and I woke up in a cell*.*” (Participant 2)*
	6	*“I have rights too*, *right*! *They can’t just wear a uniform*, *[and] abuse [us]*.*” (Participant 11)*
	7	*“There’s a little more leniency with the older police*. *But*, *the younger ones*, *just because they got a badge and a gun*, *they figure they’re somebody special*.*” (Participant 20)*
**Theme 3: Suggestions for improving experiences**		
**Subtheme**	**#**	***Participant quote***
Less judgmental police treatment	8	*“If we absolutely need them*, *we’ll call them*. *If somebody’s sitting there shot*, *half-dead*, *we’re going to call*. *We’re not stupid*. *If someone’s [overdosing]*, *we’re going to [give naloxone to] them and call 911*. *We’re not stupid*. *We don’t need six cops coming in here to help the [specific organization] staff close the [organization] at night*. *If it’s eight o’clock at night*, *we know; okay guys*, *let’s go*. *This place is closed let’s go*.*” (Participant 22)*
Police education	9	*“I’d like to see more training in post-traumatic stress disorder*.*… They feel like “I don’t have to put up with this*,*” and they are scared of the person [in need] right because the person is having a post-traumatic moment because something has triggered that trauma*.*” (Participant 1)*
	10	*“They should learn from the older police officers*.*” (Participant 5)* *“So*, *they should have them*, *like*, *well*, *[learn] how to treat a person*.*” (Participant 1)* *“More longer training*. *Because*, *like*, *when they go into the academy*, *they’re only there for a little short while*, *give them a badge and a gun*, *and put them on the streets right away*.*” (Participant 6)*
	11	*“I would love to show the police how to deal with people in the hood*. *I would do it just to show them that I ain’t whom they think I am either*.*” (Participant 4)*
Community outreach	12	*“If I could suggest*, *maybe we could have the commissioner police for Alberta do smudging once a year*, *and also*, *police could hand them out to the public in Churchill Square after they smudge*. *Under the eagle feather*.*” (Participant 6)*
	13	*“They should do a lot more of community hall information sessions*. *I know so many people who won’t go just because of the waiting room thing*. *But if you had more information about the services you’re going to get from those people*, *then maybe you will go*.*” (Participant 4)*

### Police compassion

This sub-theme denotes participants’ perception of police as supportive, caring, and friendly during encounters. One participant described such an encounter:

“Long time ago, I think I worked in [a neighbourhood in Edmonton]; at that time, there was no bus route, like every time I go by bus, I walk, and sometime they give me a ride, and they [were] asking ‘what are you doing?’ I say, ‘I finished my job; I have to go to [another neighbourhood].’ ‘Okay we will give you ride.’ Like two, three times, they gave me a ride.’

(Participant 22)

“They actually were [friendly].”

(Participant 3)

Police compassion can also save lives, as evident in another participant’s story:

“A few years ago, I was messed up, I was living on the street, I was drinking, I was using drugs, and I was very suicidal. I was walking across the High-Level Bridge, and I really wanted to look for a good place to jump because I wanted to die. Somehow or another, I ended up meeting up with a cop, and this cop was, I’ll say he was an angel because he took me out of doing what I wanted to do, and he made me realize that hey! you are a human being like everybody else, you deserve better, and he made me walk away from, without that thought of jumping.”

(Participant 10)

### People’s understanding of police work

This sub-theme denotes participants’ perception of how understanding police work and responsibilities impacted attitudes towards the police. One participant described such an impact:

“I used to hate [the police], but I came to understand that if it wasn’t for them, how bad would it really be out there? It would be everybody walking around fighting and stabbing and shooting one another. So, I do have respect for them. It’s a tough job.”

(Participant 4)

### Theme 2: Perceived unsatisfactory experiences

In contrast, some participants described having unsatisfactory experiences with the police. During these experiences, participants felt that police treatment included discrimination, stigma and disrespect, use of force, and unfair approaches.

### Discrimination

This sub-theme denotes participants’ perceptions of experiencing discrimination from the police.

Several Indigenous participants described the experience of being discriminated against based on ethnicity, as can be seen in the following conversation between two Indigenous participants:

“The police came, and they judged me right away, and they asked [my boyfriend] if I was a hooker when he met me, like who? Why would they ask him? The way I get treated from them is I get treated like shit”

(Participant 14).

“Because they’re racist. Probably 70% of the cops are racist”

(Participant 27).

### Stigma and disrespect

This sub-theme denotes participants’ perception of being disrespected by police and mistreated based on stigma. One participant experiencing housing instability described how the police interact with individuals living on the street:

“Well, what I got to say is that the police have to learn what the people on the street have to go through cause they don’t listen to them. They think just because you live on the street that you don’t know shit, you’re garbage, and you get treated like garbage.… I know most of my friends live on the street, and they respect people on the street, but the police don’t.”

(Participant 23)

### Use of force

This sub-theme denotes participants’ experience with forceful or violent police encounters. One participant described an experience where police used physical force:

“[I] remember [that] cop that used to pick on me … he was always trying, he was always using the baton, that thing they use.”

(Participant 24)

Two participants conversed about how police may forcefully interact with women:

“So far, it’s only been positive with the cops, and they’ve been nice to me, but seeing with other women, they’re actually pretty rough with them, and they beat them up.”

(Participant 5)

“Yeah, they cuff them up pretty harshly. That’s because women fight a lot better than men.”

(Participant 1)

“They molest them, and the women don’t say anything.”

(Participant 5)

“Yeah. They won’t be listened to.”

(Participant 1)

In another discussion, one participant described experiencing the use of force and felt the use was justified, but another participant criticized the use of force as unfair under certain conditions, such as with teenagers who are intoxicated (Table 3, #4). Another participant described going unconscious due to police using force on his head, as well as being pepper-sprayed (Table 3, #5). Other participants expressed concerns about the power police may assume they should have over individuals, especially the younger police officers (Table 3, #6).

### Unfair approaches

This sub-theme denotes participants’ perceptions of police engaging with or approaching individuals in an unfair way or without good reason. A participant described one such interaction:

“On their [the police] car, it says: to serve and protect. And if you’re Native, we call it a taxi. Because usually we’re being hauled off. And some guys got beaten pretty bad, and like, me and my ex-wife, we were walking through the back alley, and the next thing, you know, I’m in the back of a car, and they’re giving me all kinds of questions, and I said, why don’t you look at the cameras? There are cameras everywhere around here. So, they did that, and they released me, and the security guard that was there, he says, you must have done something wrong, and he banned me from the lot for a year.”

(Participant 35)

### Theme 3: Suggestions for improving experiences

Some participants identified factors that could improve experiences with the police. These factors included less judgemental police treatment, police education, communication style, and community outreach.

### Less judgmental police treatment

This sub-theme denotes participants’ perception that the police need to be less suspicious of and more understanding towards individuals who are often stigmatized, such as people who experience socioeconomic disadvantages. Two participants experiencing housing instability described how gathering near buildings was judged by the police:

“It’s true, though, because down here, they arrest people for lounging outside [some buildings]. Would you rather have them sitting outside of [a specific organization supporting people experiencing socioeconomic disadvantages] and drinking that case of beer where they’re not harming a goddamned person, or walking up and down the street or driving up and down the street or staggering in front of cars?”

(Participant 1)

“Exactly. If one of us community members sees someone who is overly intoxicated, we will stop and take care of that person.”

(Participant 2)

Another participant felt the police should trust people experiencing housing instability to keep an eye out and care for each other without unprompted intervention (Table 3, #8).

### Police education

This sub-theme denotes participants’ perceptions that educating the police on how respectfully engaging with individuals who are often discriminated against or stigmatized would lead to more positive experiences. One participant discussed the need to educate the police institution as a whole:

“If they didn’t start from a prejudicial point of view. Like if you’re that prejudicial and assuming when you meet someone, I don’t know how can you change it, especially if all your coworkers are the same.”

(Participant 21)

Some participants specifically suggested that the police need to be trained to help people with post-traumatic stress disorder (Table 3, #9). Others suggested having new officers train more extensively with older, more experienced police (Table 3, #10). Having the police trained by the community members they serve, such as people experiencing socioeconomic disadvantages, was also suggested (Table 3, #11).

### Communication style

This sub-theme denotes participants’ perception that police should improve communication efforts and listen more attentively. In the following conversation, two participants specifically wanted police to listen more to individuals directly involved in the situations being dealt with:

Participant 3: “They [should] try to listen to you, what’s wrong with. … Instead of other people butting in telling, oh! this is what happened. It didn’t happen to them; it happened to [me].”

The moderator: “So [they should] take the time to listen to your side.”

Participant 5: “Yeah”.

### Community outreach

This sub-theme denotes participants’ perception that the police should be more involved with the community and more intentional with sharing information about police work. One participant described an encounter where police shared information:

“I was in [a faith-based rehab program], and police came for a little visit, and they were speaking, and they were telling us how to access them. You know, just telling us about what they do, and it was kind of interesting.”

(Participant 2)

One participant (Table 3, #12) suggested that police should actively engage with the community and invited the police to participate in local Indigenous smudging ceremonies, which are traditional healing ceremonies that include burning herbs such as cedar and tobacco.[49]

Another suggested that the police should organize more information sessions about police work to improve attendance (Table 3, #13).

## Discussion

This study explored the experiences with EPS of people who experience socioeconomic disadvantages and identified factors that can impact satisfaction with policing. The present study shows that satisfaction levels may vary and identifies several factors that can be associated with them.

### Satisfactory experiences with police

The findings show that police compassion and responsiveness and people’s understanding of police work are associated with satisfactory experiences, which supports prior evidence that police compassion [50, 51] and responsiveness [19] contribute to satisfaction with police. Compassion and responsiveness can improve policing outcomes such as crime rates [11]. Compassion can be psychologically rewarding to police officers, but it can also have negative impacts on police officers’ wellbeing; when helping people becomes stressful and physically and emotionally exhausting, a phenomenon known as compassion fatigue [52] can affect individuals who provide services to people, including nurses, social workers, and police officers [52–54]. Police responsiveness is complex as police need to account for both the urgency of people’s needs and the availability of resources [55]. Police training and knowledge can also affect police responsiveness [56]. There have been some calls by grassroots organizations in many countries, including Canada and the USA, to reform policing by reducing police funding [57, 58]; however, evidence suggests that this might impede police responsiveness [59]. Understanding police work was also associated with satisfaction with police. Education regarding police capabilities and capacities may help people better understand and evaluate police responsiveness. As such, police services may need to develop more sophisticated methods to communicate with the public regarding how police calls are prioritized based on available resources [60, 61]. Educating people about laws, especially youth, might increase compliance with laws [62]. Police services might benefit from collaborating with schools and community organizations to reach to different population groups to educate people about police work [62].

### Unsatisfactory experiences with police

This study found that discrimination, stigma, disrespect, use of force, and unfair approaches from police resulted in unsatisfactory experiences. These factors of policing are antithetical to the principles of procedural justice and weaken the perceived legitimacy of police services [11]. As such, unsatisfactory interactions with the police may prevent certain individuals from seeking emergency help, which has implications for public health.

Discrimination from the police is common in many countries, including Canada [9, 63]. Indigenous communities are disproportionately subjected to racial profiling and carding in Canada [9, 63] and are also more likely to have involuntary contact with police, which is a strong predictor of people losing confidence in the police [64, 65]. Several reports and studies revealed discrimination against Indigenous community members by the police [66]. The relationship between Indigenous communities and the police may also be influenced by historical colonial practices, in which the police was a major law enforcement instrument; these practices exposed Indigenous communities to systemic social inequities and inter-generational trauma, resulting in disproportionate and frequent encounters with police as law violators or victims [66–68]. Similar experiences with the police, characterized by loss of confidence and dissatisfaction with police performance and fairness, have also been reported in Black Canadian communities [65]. In a recent study examining data from 1994 to 2019 the majority of Black Canadians in Toronto noted that the police force is racially discriminative [69]. Another study found that Black Canadians and Indigenous community members are more likely to be arrested for drug possession cases than White Canadians [70].

Stigma against people experiencing housing instability was also found in this study to be a factor of unsatisfactory police experiences. Research in the UK, USA, and Canada similarly suggests that police interactions with individuals experiencing housing instability are often characterized by stigmatization, frequent identity-checking, mistreatment, and harassment [71, 72].

The use of force, specifically with intoxicated youth, was also found to be a factor in having unsatisfactory experiences. Evidence suggests that situational conditions, including suspect’s resistance, intoxication, weapon use, and an officer being alone, strongly affect the likelihood and level of using force by police [73, 74]. Other factors that might be associated with using force include neighbourhood crime rates, the ethnicity of the suspect, and the police department’s training and use of force policies [75, 76]. The use of force negatively affects the community-police relationship and is recognized as an important public health issue [77, 78] that also contributes to racial health inequity [79]. Research from Canada and the USA suggests that intoxicated youth experience police violence [74, 75], resulting in physical injuries and potentially impacting psychological health, which can have long-term effects [80, 81]. Participants in this study also reported inappropriate use of force toward women and that there may be a concerning power imbalance between police and the community members they serve. In Canada, the use of force is governed by the Criminal Code, which permits the use of force by police officers when there are “reasonable grounds that the force is necessary” for self-protection or the protection of other people under threat [82]. However, there are intensifying concerns about the increasing use of force by the police in Canada [83], including concerns about the use of force with ethnic minorities in particular [83] and the scarcity of data about the use of force incidents and police training [83, 84].

The relationship between intoxicated people and people experiencing housing instability and the police in Canada might be affected by federal, provincial, and municipal laws. The Criminal Code of Canada prohibits loitering in public places and causing any form of disturbance near people’s properties [82]. Public consumption of alcohol is prohibited in Alberta except in designated areas in parks, and the police may arrest intoxicated persons [85]. Aggressive panhandling is prohibited in Edmonton, including obstructing people’s passage, insulting or threatening people, or being intoxicated [86]. Therefore, the frequent interactions between the police and intoxicated people or people experiencing housing instability might be legitimate from the police’s perspective but still be unsatisfactory to people. As presented in this study, adherence to the procedural justice principles can help reduce people’s dissatisfaction.

### Improving people’s experiences with police

Study participants made several suggestions to improve people’s experiences with police. These are police being less judgemental, receiving education, improving communication, and implementing more community outreach,. Communication is critical to achieving procedural justice and maintaining trust in the police [11]. Police’s respectful communication during encounters increases people’s satisfaction [87–89]. As well, educating police on topics including cultural sensitivity and procedural justice may positively impact policing interactions and influence police decision-making [88–90]. Educating police further on human rights and socioeconomic issues, including housing instability and food insecurity, could also positively impact the community-police relationship [36, 91].

Participants’ suggestions support recent EPS initiatives to improve the policing experience, build trust and better understand the city’s different communities [92]. EPS created the Commitment to Action (C2A) plan in September 2020, which aims to build a city that feels safer for all residents. The plan has facilitated listening sessions, engaging a diverse cross-section of the city through online forums and surveys [93]. The C2A plan has identified six priorities for the EPS: relationship building, partnership development, training and professional development, communication and transparency, innovation, and community engagement. Our study findings reinforce these six priorities. EPS initiatives include the development of experiential learning modules with community partners to help police recruits build trust and gain a better understanding of the communities they serve. Learning modules include trauma-informed policing and experiences of Indigenous, 2SLGBTQ+, and newcomers [94], and a community practicum placement in community organizations serving people experiencing socioeconomic disadvantages [95]. EPS has developed bias awareness training, which is mandatory for all its members [96]. EPS officers also receive training on community policing, procedural justice, cultural safety, and trauma-informed policing [96]. These initiatives are expected to improve people’s satisfaction and relationship with the police; however, data on the outcomes of these initiatives are limited. As well, police training should be continuous and accompanied by performance monitoring to ensure that expected educational outcomes are achieved and that the training is effective in improving policing and people’s experience with the police [40]. Another call to improve policing came from the Edmonton City Council in 2021, which suggested reducing police presence in non-crime situations, reducing the use of force, adding social workers and mental health professionals to response teams when necessary, eliminating discriminatory practices, and tying part of this funding to police ability to improve police education [97].

### The importance of the present study

This study is timely considering the increasing calls for policing reforms worldwide. It contributes to the growing body of research exploring policing experiences and the police-community relationship, both of which can impact the health and wellbeing of individuals. The study provides empirical evidence for policymakers and researchers to use in designing policy interventions or future research projects.

Although the study only explored policing experiences and did not address health outcomes of policing directly, it uses existing evidence to assert the strong relationship between those experiences and health outcomes. Given the qualitative design of the study, its findings cannot be transferred without considering the contextual factors and the characteristics of different population groups.

## Conclusions

Various factors influence the community-police relationship. This study shows that police responsiveness and compassion and improved people’s understanding of police operations led to satisfactory interactions. On the other hand, police practices, such as discrimination, stigma, disrespect, use of force, and unfair approach from police, can lead to unsatisfactory interactions and may harm procedural justice and weaken the perceived legitimacy of police services.

As policing is now being understood as a determinant of health, the police force needs to minimize unsatisfactory experiences through less judgemental policing and improved police communication and community engagement. Practically, police education is paramount to improving policing and community-police relationships. Police education should cover procedural justice, cultural sensitivity, human rights and socioeconomic issues, including housing instability and food insecurity. Additionally, the reform initiatives presented in this paper should be continually and collaboratively evaluated by the police, the community, and other stakeholders to ensure accountability.

## Supporting information

S1 Table Consolidated criteria for reporting qualitative studies (COREQ): 32-item checklist. (DOCX)
