# Supplementary material for: Community experiences with police and implications for public health: A focus group study
Source: PLOS Glob Public Health. 2024 Jun 11;4(6):e0003123. doi: 10.1371/journal.pgph.0003123 (PMC11166326; doi:10.1371/journal.pgph.0003123)
Supplement: S1 Table — (DOCX) [file pgph.0003123.s001.docx]

**Community experiences with police and implications for public health: a focus group study.**

**S1 Table.** Consolidated criteria for Reporting Qualitative studies (COREQ): 32-item checklist

| **No** | **Item** | **Guide questions/description** | **Page/Section/ /Paragraph** |  |
| --- | --- | --- | --- | --- |
| **Domain 1: Research team and reflexivity** | | | | |
| Personal Characteristics | | | | |
| 1. | Interviewer/facilitator | **Which author/s conducted the interview or focus group?** | Page 5/Methods/ Paragraph 4 |  |
| 2. | Credentials | **What were the researcher's credentials? *E.g. PhD, MD***  [SJ] is a PhD student.  [MZ], [FK], [SD], [CG], and [SS] have PhD degree.  [AW] MD  All researchers have extensive experience in qualitative research and public health. | This table. |  |
| 3. | Occupation | **What was their occupation at the time of the study?**  [MZ], [SD], and [CG] were PhD students.  [FK] was executive director of the Indigenous and Global Health Research Group at the University of Alberta.  [SJ] was a research assistant and had a bachelor’s degree in nutrition.  [SS] was a professor in Indigenous and Global Health Research at the at the University of Alberta.  [AW] was a professor in Geriatric Medicine at the University of Alberta | This table. |  |
| 4. | Gender | **Was the researcher male or female?**  [MZ] and [AW] are males.  [SJ], [FK], [SD], [CG], and [SS] are females | This table. |  |
| 5. | Experience and training | **What experience or training did the researcher have?**  [MZ] received graduate level training on qualitative research, and has conducted several qualitative research studies on population health, patient experience, and healthcare providers’ perspectives.  [SJ] received graduate level training on qualitative research and participated in several qualitative research studies on Indigenous people’s health, patient experience, and healthcare providers’ perspectives.  [FK] received graduate level training on qualitative research, and has conducted several qualitative research studies on population health, Indigenous people’s health, patient experience, and healthcare providers’ perspectives.    All the other researchers bring extensive experience in qualitative research and health research in general and have worked on research on health of people experiencing socioeconomic disadvantages. | This table. |  |
| Relationship with participants | | | | |
| 6. | Relationship established | Was a relationship established prior to study commencement? | Page 5/Methods/ Paragraph 2 |  |
| 7. | Participant knowledge of the interviewer | What did the participants know about the researcher? e*.g. personal goals, reasons for doing the research* | Page 5/Methods/ Paragraph 2 |  |
| 8. | Interviewer characteristics | What characteristics were reported about the interviewer/facilitator? e.g. *Bias, assumptions, reasons and interests in the research topic* | Page 6/ Methods/Paragraph 2 |  |
| **Domain 2: study design** | | | | |
| Theoretical framework | | | | |
| 9. | Methodological orientation and Theory | What methodological orientation was stated to underpin the study? *e.g. grounded theory, discourse analysis, ethnography, phenomenology, content analysis* | Page 4/Methods/ Setting, design and data collection/ Paragraph 1 |  |
| Participant selection | | | | |
| 10. | Sampling | How were participants selected? *e.g. purposive, convenience, consecutive, snowball* | Page 4/Methods/ Setting, design and data collection/Paragraph 2 |  |
| 11. | Method of approach | How were participants approached? e*.g. face-to-face, telephone, mail, email* | Methods/Page 5/ Paragraph 2 |  |
| 12. | Sample size | How many participants were in the study? | Page 5/Methods/ Paragraph 6 |  |
| 13. | Non-participation | How many people refused to participate or dropped out? Reasons? | Page 5/Methods/ Paragraph 2 |  |
| Setting | | | | |
| 14. | Setting of data collection | Where was the data collected? e*.g. home, clinic, workplace* | Page 6/Methods/ Paragraph 1 |  |
| 15. | Presence of non-participants | Was anyone else present besides the participants and researchers?   No |  |  |
| 16. | Description of sample | What are the important characteristics of the sample? *e.g. demographic data, date* | Page 6/Methods/ Paragraph 1  And  Page 8/Results / Paragraph 1 |  |
| Data collection | | | | |
| 17. | Interview guide | **Were questions, prompts, guides provided by the authors? Was it pilot tested?**  Questions were provided but they were not pilot tested. | Page 6/Methods/ Paragraph 2 |  |
| 18. | Repeat interviews | **Were repeat interviews carried out? If yes, how many?**  No |  |  |
| 19. | Audio/visual recording | Did the research use audio or visual recording to collect the data? | Page 6/Methods/ Paragraph 3 |  |
| 20. | Field notes | Were field notes made during and/or after the interview or focus group? | Page 6/Methods/ Paragraph 2 |  |
| 21. | Duration | What was the duration of the interviews or focus group? | Page 6/Methods/ Paragraph 1 |  |
| 22. | Data saturation | Was data saturation discussed? | Page 6/Methods/ Paragraph 3 |  |
| 23. | Transcripts returned | **Were transcripts returned to participants for comment and/or correction?**  No |  |  |
| **Domain 3: analysis and findings** | | | | |
| Data analysis | | | | |
| 24. | Number of data coders | **How many data coders coded the data?**  Two researchers did the analysis.  Results were verified independently by the other authors. | Page 7/Methods/data analysis/ Paragraph 1 |  |
| 25. | Description of the coding tree | Did authors provide a description of the coding tree? | Page 7/Methods/data analysis/Paragraph 1 |  |
| 26. | Derivation of themes | **Were themes identified in advance or derived from the data?**  All themes were identified in advance. | Page 7/ Methods/data analysis/Paragraph 1 |  |
| 27. | Software | What software, if applicable, was used to manage the data? | Page 7/ Methods/data analysis/Paragraph 1 |  |
| 28. | Participant checking | **Did participants provide feedback on the findings?**  No |  |  |
| Reporting | | | | |
| 29. | Quotations presented | **Were participant quotations presented to illustrate the themes / findings? Was each quotation identified?** e*.g. participant number*  Yes | Pages 9 to 17/Results |  |
| 30. | Data and findings consistent | **Was there consistency between the data presented and the findings?**  Yes. Quotations were carefully selected. | Pages 9 to 17/Results |  |
| 31. | Clarity of major themes | Were major themes clearly presented in the findings? | Pages 9, 13, 15/Results |  |
| 32. | Clarity of minor themes | **Is there a description of diverse cases or discussion of minor themes?**  Yes. Sub-themes were presented under the main themes | Pages 9, 13, 15/Results |  |
